# Cytokines Removal During *Ex-Vivo* Lung Perfusion: Initial Clinical Experience

**DOI:** 10.3389/ti.2023.10777

**Published:** 2023-08-14

**Authors:** Massimo Boffini, Matteo Marro, Erika Simonato, Fabrizio Scalini, Andrea Costamagna, Vito Fanelli, Cristina Barbero, Paolo Solidoro, Luca Brazzi, Mauro Rinaldi

**Affiliations:** ^1^ Cardiac Surgery Division, Surgical Sciences Department, Città della Salute e della Scienza, University of Turin, Turin, Italy; ^2^ Anesthesiology and Intensive Care Division, Surgical Sciences Department, Città della Salute e della Scienza, University of Turin, Turin, Italy; ^3^ Pulmonology Division, Medical Sciences Department, Città della Salute e della Scienza, University of Turin, Turin, Italy

**Keywords:** lung transplant, *ex-vivo* lung perfusion, ischemia-reperfusion, cytokines, inflammation, primary graft dysfunction

## Abstract

*Ex Vivo* Lung Perfusion (EVLP) can be potentially used to manipulate organs and to achieve a proper reconditioning process. During EVLP pro-inflammatory cytokines have been shown to accumulate in perfusate over time and their production is correlated with poor outcomes of the graft. Aim of the present study is to investigate the feasibility and safety of cytokine adsorption during EVLP. From July 2011 to March 2020, 54 EVLP procedures have been carried out, 21 grafts treated with an adsorption system and 33 without. Comparing the grafts perfused during EVLP with or without cytokine adsorption, the use of a filter significantly decreased the levels of IL10 and GCSFat the end of the procedure. Among the 38 transplanted patients, the adsorption group experienced a significant decreased IL6, IL10, MCP1 and GCSF concentrations and deltas compared to the no-adsorption group, with a lower in-hospital mortality (*p* = 0.03) and 1-year death rate (*p* = 0.01). This interventional study is the first human experience suggesting the safety and efficacy of a porous polymer beads adsorption device in reducing the level of inflammatory mediators during EVLP. Clinical impact of cytokines reduction during EVLP must be evaluated in further studies.

## Graphical Abstract

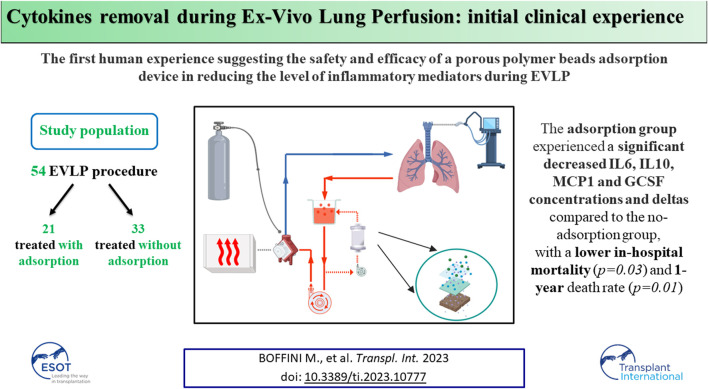

## Introduction

Lung transplantation (LTx) is a well-established therapy for selected patients with various forms of end stage, progressive lung disease. Since the first lung transplant in 1963, the field of LTx has advanced in the selection of candidates, operative techniques, critical care management, immunosuppression, and long-term follow-up. During the last 10 years a significant increase of lung transplant procedures has been recorded if compared with other organs [1]. According to the 2020 International Society for Heart and Lung Transplantation (ISHLT) Registry, almost 70,000 adult lung transplant procedures have been reported since its inception [2]. However, a significant imbalance between the number of transplants performed and the clinical demand still remains.

Although nowadays LTx is a well-established treatment for patients with end-stage lung diseases, shortage of suitable lung grafts is still a major limitation for an extensive application of this therapy. Mortality of patients on the waiting list for a lung transplant is the highest if compared with other solid organ transplants and it can be estimated that up to one out of five patients on the lung transplant waiting list will die before a suitable organ is identified. A major challenge facing the lung transplant community is how to increase the number of usable donor lungs without compromising the success of the procedure. Lungs from donors both after brain death (BDD) and cardiac death (DCD) are subjected to several injurious mechanisms during the donation process. Attempt to transplant injured donor lungs can lead to high incidence of severe primary graft dysfunction (PGD) and associated short- and long-term poorer results [3]. Thus, it is not surprising that the majority of donor lungs are not utilized for transplantation and among the donor pool, the utilization rate of lung grafts is nearly 20%. Expansion of the donor pool has been attempted by extending the conventional donor selection criteria, by use of DCD and, lastly, with the implementation of *Ex Vivo* Lung Perfusion (EVLP) techniques. The ideal donor characteristics are the followings: age <55 years, with a smoking history <20 pack-year, no chest trauma, clear chest X-ray, PaO_2_/FiO_2_ (P/F) ratio >300 and absence of purulent secretions at bronchoscopy [4]. This scenario is known to correspond to less than half of the donors utilized for transplantation [5] and what have previously been regarded as “ideal” donor lung criteria by the ISHLT are becoming less representative of what is now deemed acceptable in most centers. This has raised the numbers of available donor lungs for transplant, but this may increase the complexity of clinical management of the transplanted patients [6]. EVLP has emerged as a relatively novel technique for preserving, evaluating and eventually reconditioning extended criteria donor lungs. Lung transplant activity may be increased by 15%–30% in Lung Transplant Programs adopting EVLP protocols [7, 8].

Ischemia-reperfusion (IR) injury after lung transplantation can lead to devastating complications, such as primary graft dysfunction, acute rejection, chronic graft dysfunction with a significant impact on morbidity and mortality [9, 10]. In the setting of IR injury, cytokine production plays a crucial role in mediating the inflammatory process that can leave the donor lung permanently dysfunctional. Cytokines and chemokines are small molecules that promote injury through neutrophil recruitment, capillary leakage and cell death [11]. Pro-inflammatory cytokines have been shown to accumulate progressively in perfusate over time during EVLP and cytokine increase has been correlated with poor outcomes related to PGD [12]. CytoSorb^®^ is an absorbent filter which is highly effective in non-selective but concentration-dependent removal of mediators between the molecular weight of 10 and 50 kDa through a 850 m^2^/g surface.

Aim of the present study is to investigate the feasibility and safety of the adsorbent filter CytoSorb^®^ during EVLP.

## Materials and Methods

### Study Design

In July 2011 a reconditioning program based on EVLP has been activated at the Lung Transplant Center of Città della Salute e della Scienza University Hospital in Turin, Italy. Until March 2020, 54 perfusions have been carried out on pulmonary grafts deemed unacceptable for direct transplantation at donation site. Among those, 38 (70.4%) grafts showed a normal function after perfusion and they have been transplanted (31 bilateral and 7 single LTx). EVLP program allowed a nearly 30% of increase of lung transplant activity and LTx using perfused grafts is the 22% of all LTx performed in Turin. The reconditioning protocol is that described by the Toronto Lung Transplant Group [3] and perfusion has been conducted for 4–6 h. Very briefly, our protocol is based on four principles: 1) use of an acellular solution (STEEN Solution), 2) closed circuit allowing a constant positive pressure in the left atrium, 3) low flow perfusion (with a target flow of 40% of theoretical cardiac output), 4) protective ventilation (tidal volume 7 mL/kg of donor’s predicted body weight, respiratory rate 7/min, positive end expiratory pressure 5 cm H_2_O, Fraction of Inspired Oxygen, FiO_2_, 21%). Preliminary clinical results have been already published elsewhere [13].

**FIGURE 1:**
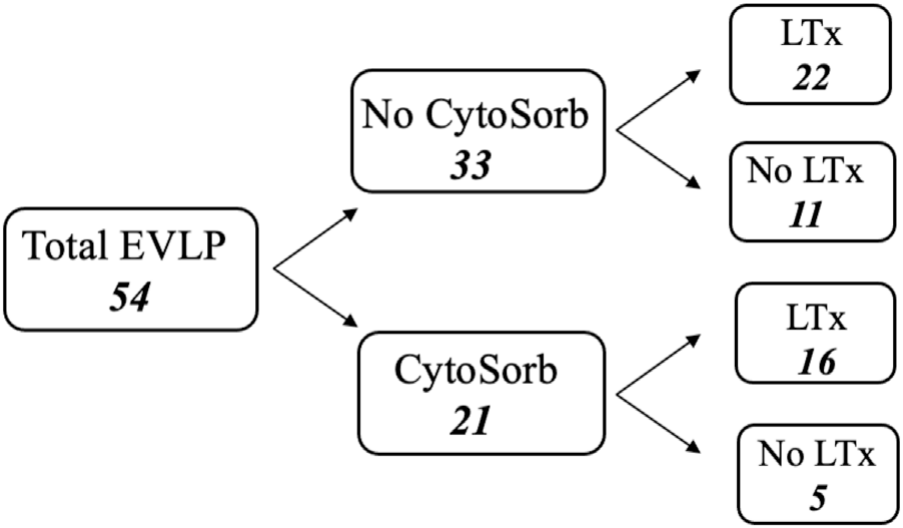
Flow chart of total *ex-vivo* lung perfusion procedures.

EVLP has been accomplished using components available for every day clinical practice. An Euroset™ circuit with Admiral oxygenator, an anti-leucocyte filter (Pall LeukoGuard-6^®^ Arterial Filter) and a Medtronic Bio-Medicus^®^ pump have been used for perfusion and the circuit has been connected to the graft through specially designed funnel-shaped cannulas with built-in pressure probes (Vitrolife^®^). The circuit has been primed with a buffered extracellular solution added with an optimal colloid osmotic pressure and dextran (Steen Solution™), broad-spectrum antibiotics (imipenem/cilastatin 500 mg/500 mg), heparin (10,000 IU) and methylprednisolone (500 mg). In two cases burdened with significant pulmonary embolism, fibrinolytic agents have been added to the perfusate. From October 2016 the last 21 consecutive grafts have been perfused adding CytoSorb^®^ filter to the circuit. CytoSorb^®^ has been connected via a veno-venous shunt from the reservoir, filtering the perfusate which is then re-collected in the reservoir. Among these, 16 (76%) grafts have been transplanted. Out of the 33 grafts treated without CytoSorb^®^ system, 22 (67%) have been transplanted (Figure 1).

**FIGURE 2:**
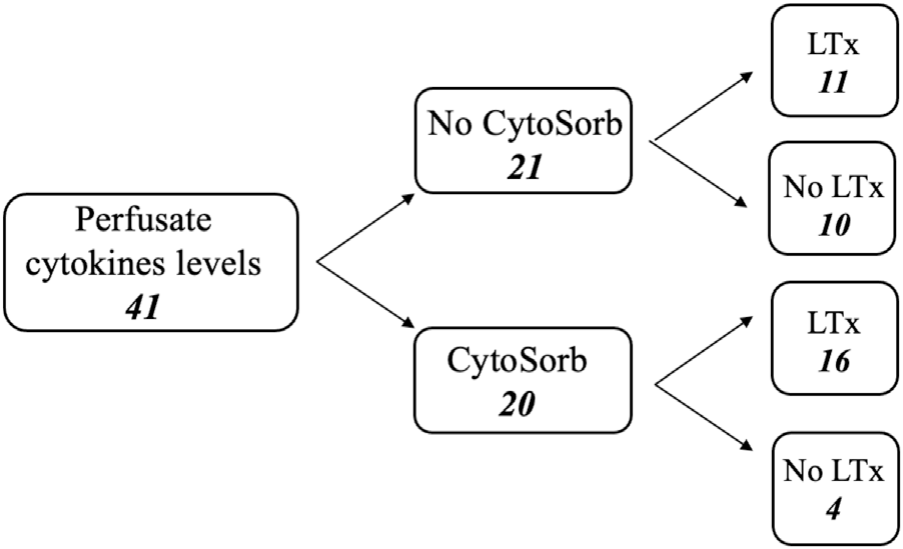
Flow chart of perfusate analysis during *ex-vivo* lung perfusion procedures.

The cytokines levels in the perfusate (interleukin 10/IL-10, interleukin 6/IL-6, monocyte chemotactic protein 1/MCP-1 and granulocyte colony-stimulating factor/G-CSF) at the beginning (time 0, *T0*) and at the end of the EVLP (final time, *Tf*) have been measured in 41 procedures and the results have been analyzed. Figure 2 shows a flow chart.

Functional assessment of EVLP was performed hourly. Dynamic compliance was calculated as the ratio between tidal volume and delta pressure (= peak inspiratory pressure minus positive end-expiratory pressure); static compliance was calculated as the ratio between tidal volume and delta pressure (= plateau pressure minus positive end-expiratory pressure). Blood gas tests were performed on perfusate samples to calculate the left atrial PO_2_.

The protocol was created in adherence to the Institutional Review Board of *Città della Salute e della Scienza di Torino* (IRB: 2CEI-178).

### Inclusion and Exclusion Criteria for EVLP

#### Donors

High-risk donor lungs were defined as those meeting any of the following criteria: P/F ratio of less than 300 mmHg; multiple blood transfusions; pulmonary edema detected by chest X-ray or by bronchoscopy or surgical evaluation. Donor lungs with diagnosed pneumonia, persistent secretions on bronchoscopy, aspiration, trauma or contusion were excluded. Pulmonary embolism was not considered a contra-indication and two grafts with severe pulmonary embolism have been perfused with Steen Solution and fibrinolytic agents before their implant.

#### Recipients

All patients awaiting a single or bilateral lung transplant at Città della Salute e della Scienza University Hospital in Turin who have given written informed consent to transplantation with a reconditioned graft, were eligible. After the EVLP, the graft was transplanted if the following parameters were achieved: delta PaO_2_ (PaO_2_ in the pulmonary veins–PaO_2_ in the pulmonary artery) higher than 350 mmHg; stability or improvement of organ hemodynamic parameters (pulmonary artery pressure ≤15 mmHg, pulmonary vascular resistance stable or decreased) lung dynamics (stable or increased static and dynamic compliance, stable or decreased airway pressure); lung X-ray and bronchoscopy negative; positive clinical judgment of the transplant team.

### Statistical Analysis

Data were tested for normal distribution by Shapiro-Wilk test and a test on the equality of standard deviations (variances) on every variable was performed. Data were expressed as mean and standard deviation (SD) or median with interquartile range 25–75 (IQR), as appropriate.

Base 10 logarithmic transformations on absolute cytokine’s levels (IL-6, IL-10, GCSF and MCP1) were performed to reduce skewness and kurtosis.

Descriptive statistics are presented as mean, median, standard deviation and ranges for the continuous variables, and as counts and percentages for categorical variables. Differences between groups were assessed with Wilcoxon rank-sum test for independent samples and Wilcoxon matched-pairs signed-rank test for matched pairs and with t tests (paired or unpaired) on the equality of means as appropriate. Categorical variables were analyzed with Chi-squared or Fisher’s exact test, as appropriate. Statistical difference has been considered significant for *p* < 0.05. All analyses were performed using Stata 16.1/SE (Stata Corp TX, United States) and SPSS 20.0 (IBM Corp., Armonk, NY, United States).

## Results

Cytokines levels at *T0* and *Tf* and deltas (difference between *T0* and *Tf*) are described in details in Tables 1, 2.

**TABLE 1:** Comparison of cytokines concentration in the perfusate at the beginning (*T0*) and at the end (*Tf*) of *ex-vivo* lung perfusion with and without CytoSorb^®^.

	No-Cytosorb (*n* = 21)	Cytosorb (*n* = 20)	*p*-value
IL-6 _log10_ T0	3.0 ± 1.7	3.5 ± 0.7	0.8708
IL-6 _log10_ Tf	4.8 ± 0.2*	4.4 ± 0.4*	0.0002
IL-6 _log10_ delta	1.7 ± 1.7	0.9 ± 0.6	0.1550
IL-10 _log10_ T0	1.3 ± 0.7	1.1 ± 0.5	0.2382
IL-10 _log10_ Tf	2.6 ± 0.4*	1.9 ± 0.4*	0.0000
IL-10 _log10_ delta	1.3 ± 0.7	0.8 ± 0.7	0.0440
MCP1 _log10_ T0	3.2 ± 0.6	2.6 ± 0.5	0.0020
MCP1 _log10_ Tf	3.8 ± 0.3*	3.0 ± 0.4*	0.0000
MCP1 _log10_ delta	0.6 ± 0.5	0.4 ± 0.5	0.1408
GCSF _log10_ T0	1.6 ± 1.6	1.8 ± 0.9	0.5935
GCSF _log10_ Tf	4.0 ± 0.6*	3.3 ± 0.6*	0.0015
GCSF _log10_ delta	2.4 ± 1.4	1.4 ± 0.9	0.0358

List of abbreviations: IL-6, interleukin 6; IL-10, interleukin 10; MCP1, monocyte chemotactic protein 1; GCSF, granulocyte colony-stimulating factor. _log10_, natural logarithm *p < 0.01 vs. T0.

**TABLE 2:** Comparison of cytokines concentration in the perfusate at the beginning (*T0*) and at the end (*Tf*) of *ex-vivo* lung perfusion with and without CytoSorb^®^ in transplanted grafts.

	No-Cytosorb (*n* = 11)	Cytosorb (*n* = 16)	*p-value*
IL-6 _log10_ T0	2.1 ± 1.7	3.5 ± 0.7	0.0577
IL-6 _log10_ Tf	4.8 ± 0.2*	4.3 ± 0.4*	0.0002
IL-6 _log10_ delta	2.8 ± 1.7	0.9 ± 0.7	0.0014
IL-10 _log10_ T0	1.1 ± 0.5	1.1 ± 0.5	0.9172
IL-10 _log10_ Tf	2.7 ± 0.4*	1.9 ± 0.3*	0.0000
IL-10 _log10_ delta	1.6 ± 0.5	0.8 ± 0.6	0.0027
MCP1 _log10_ T0	3.0 ± 0.5	2.6 ± 0.6	0.0709
MCP1 _log10_ Tf	3.8 ± 0.2*	3.0 ± 0.4**	0.0000
MCP1 _log10_ delta	0.8 ± 0.4	0.4 ± 0.6	0.0229
GCSF _log10_ T0	0.7 ± 1.1	1.7 ± 0.9	0.0242
GCSF _log10_ Tf	3.8 ± 0.6*	3.3 ± 0.6*	0.0457
GCSF _log10_ delta	3.1 ± 0.8	1.4 ± 1.0	0.0002

List of abbreviations: IL-6, interleukin 6; IL-10, interleukin 10; MCP1, monocyte chemotactic protein 1; GCSF, granulocyte colony-stimulating factor; _log10_, natural logarithm; *p < 0.01 vs. *T0*; **p < 0.05 vs. *T0*.

**FIGURE 3:**
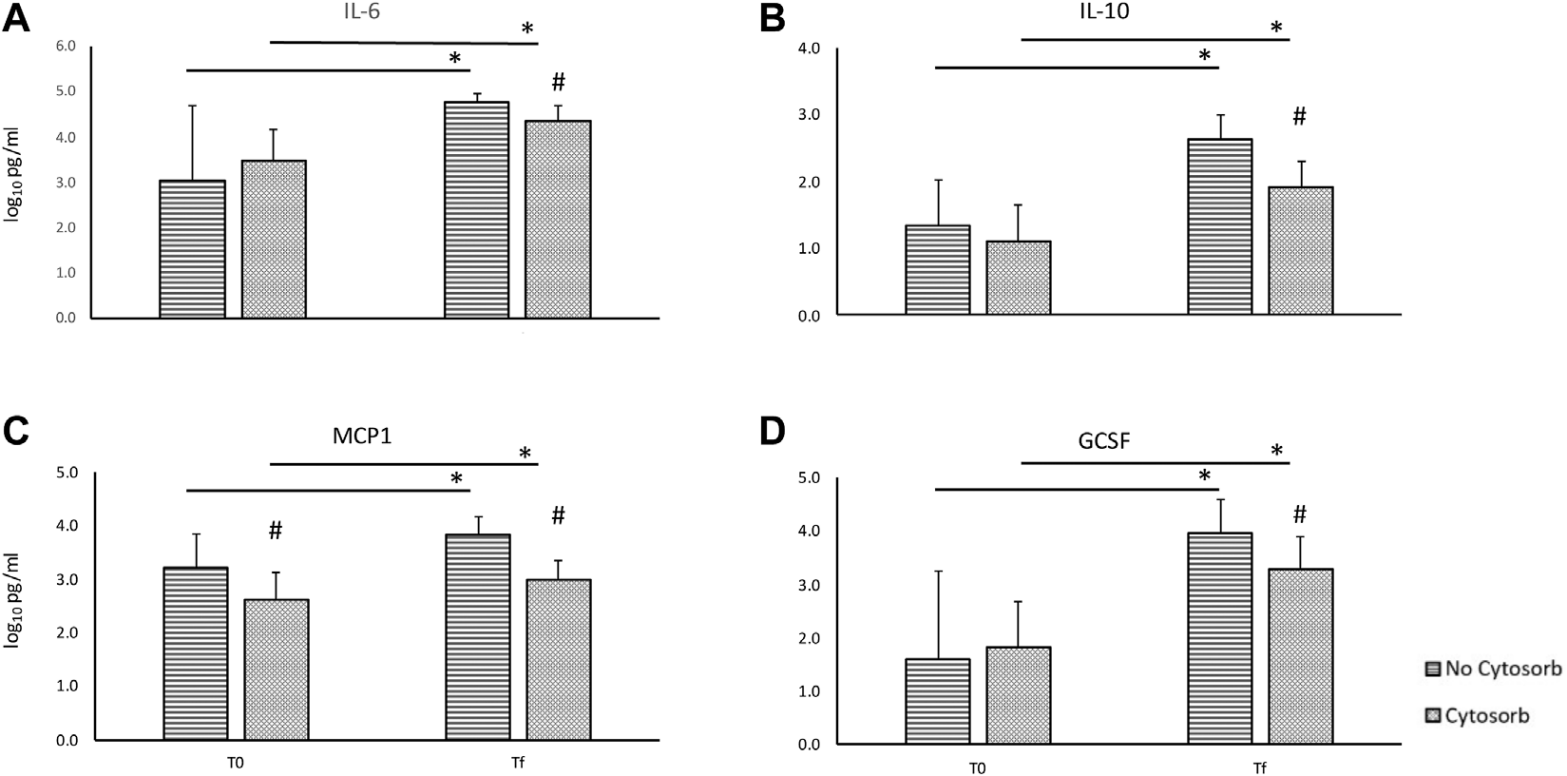
Comparison of cytokines concentration in the perfusate at the beginning (*T0*) and at the end (*Tf*) of *ex-vivo* lung perfusion with and without CytoSorb^®^ [base 10 log of IL6, panel **(A)**; base 10 log of IL10, panel **(B)**; base 10 log of MCP1, panel **(C)**; base 10 log of GCSF, panel **(D)**]. Values are expressed as mean ± standard deviations. *p < 0.01 vs. T0; **p < 0.05 vs. T0; ^#^ *p* < 0.01 vs. no-Cytosorb group; ^##^ *p* < 0.05 vs. no-Cytosorb group.

**FIGURE 4:**
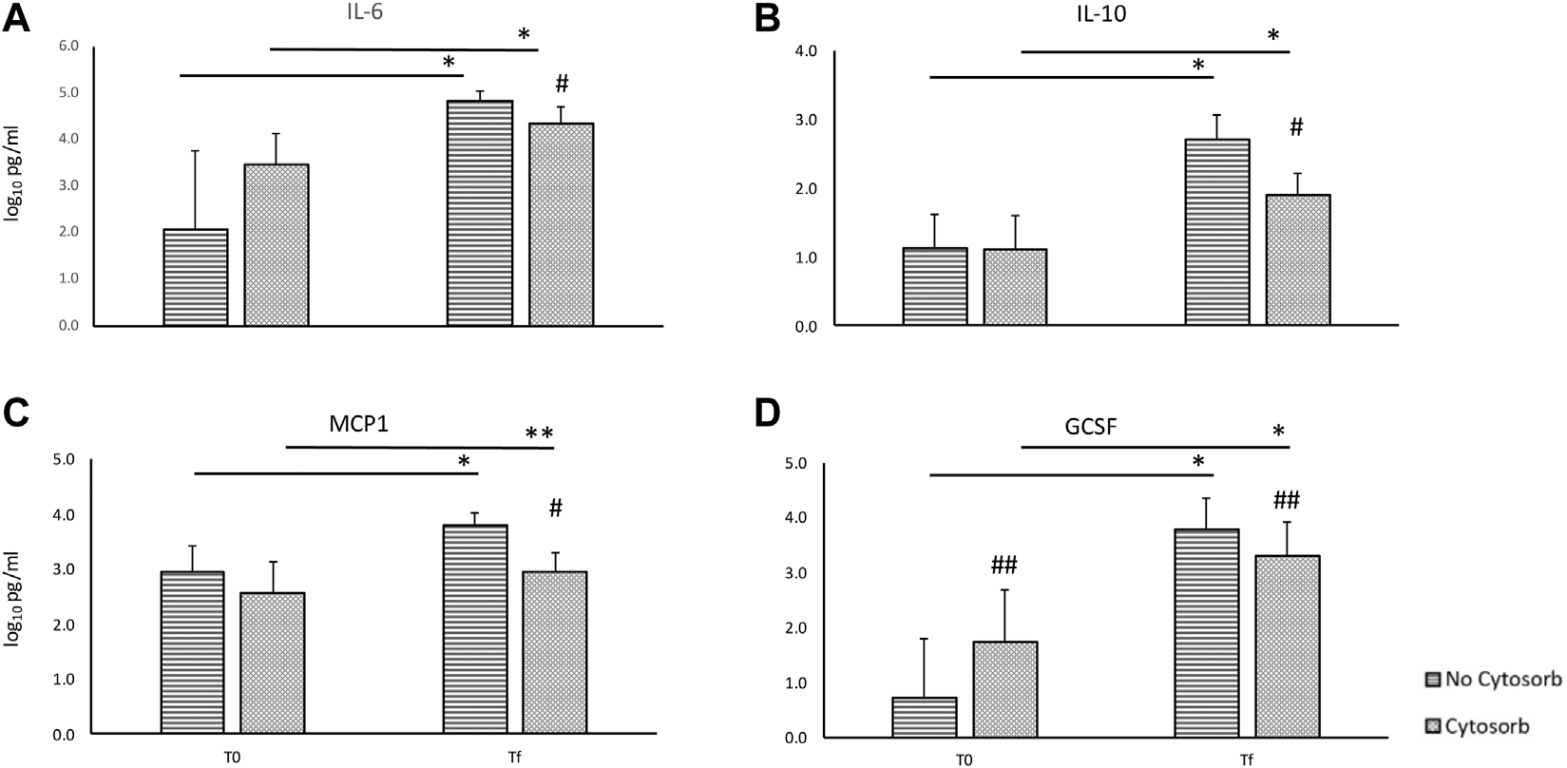
Comparison of cytokines concentration in the perfusate at the beginning (*T0*) and at the end (*Tf*) of *ex-vivo* lung perfusion with and without CytoSorb^®^ in transplanted grafts [base 10 log of IL6, panel **(A)**; base 10 log of IL10, panel **(B)**; base 10 log of MCP1, panel **(C)**; base 10 log of GCSF, panel **(D)**]. Values are expressed as mean ± standard deviations. *p < 0.01 vs. T0; **p < 0.05 vs. T0; ^#^ *p* < 0.01 vs. no-Cytosorb group; ^##^ *p* < 0.05 vs. no-Cytosorb group.

During EVLP, cytokines’ levels increase over time with a significant difference between *T0* and *Tf* both overall and in the transplanted group (Tables 1, 2) despite the use of Cytosorb^®^. In overall perfusions, at the comparison between the Cytosorb^®^ vs. no-Cytosorb group^®^, deltas are similar for IL6 and MCP1 (*p* = 0.15 and *p* = 0.14), and decreased only for IL10 (*p* = 0.04) and GCSF (*p* = 0.03). All the details are reported in Table 1 and Figure 3. Table 2 shows the results obtained from the comparison between the transplanted grafts perfused with or without the use of CytoSorb^®^. The two cohorts have similar levels of cytokines at *T0*, significant decreased IL6, IL10, MCP1 and GCSF concentrations and deltas in the Cytosorb^®^ group (Table 2 and Figure 4).

**FIGURE 5:**
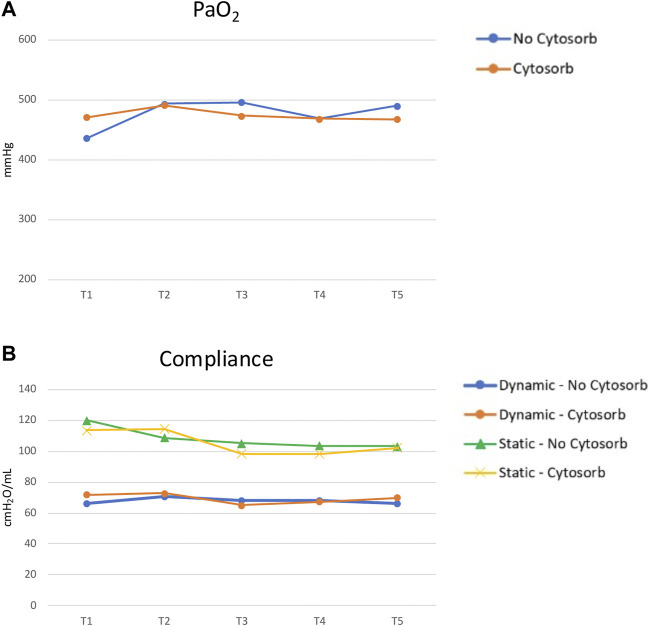
EVLP parameters comparison between Cytosorb and no-Cytosorb group in transplanted grafts [PaO_2_, panel **(A)**; static and dynamic compliance, panel **(B)**].

Among the transplanted grafts, the comparison of “physiological assessment” (based on gas exchange and lung dynamics) during EVLP stratified according to the use or not of CytoSorb^®^ suggests no difference, but a test could not be performed (Figure 5).

Table 3 summarizes the comparison of donors’ and recipients’ characteristics of transplanted grafts with or without CytoSorb^®^ during EVLP. Donors are similar in the two groups being the duration of mechanical ventilation, that is longer in the no-CytoSorb^®^ group (4 ± 2 vs. 3 ± 2 days, *p =* 0.02), the only significant difference. Patients in the CytoSorb^®^ group are older (60 [54–63] vs. 49 [35–57], *p* = 0.02), received a bilateral lung transplant and required CPB less frequently (63% vs. 95%, *p* = 0.01, 31% vs. 73%, *p* = 0.01, respectively).

**TABLE 3:** Comparison of recipient and donor characteristics in transplanted grafts after *ex-vivo* lung perfusion with and without CytoSorb^®^.

	Cytosorb (*n* = 16)	no-Cytosorb (*n* = 22)	*p-value*
Baseline LTx recipient characteristics			
LAS score	31.2 [30.8–31.3]	31.4 [30.7–31.6]	0.28
Age at transplant (years)	60 [54–63]	49 [35–57]	0.02
Male sex (*n*, %)	10 (63)	13 (59)	0.80
ECMO at LTx (*n*, %)	0 (0)	2 (9)	0.49
Bilateral lung transplantation (*n*, %)	10 (63)	21 (95)	0.01
CPB during LTx (*n*, %)	5 (31)	16 (73)	0.01
Ischemia time (min)	784 ± 96	781 ± 33	0.9
Lung disease			
Idiopatic fibrosis	8	7	
Cystic fibrosis	2	5	0.9
COPD	4	6	
Vascular disease	1	3	
Other	1	1	
Baseline donor characteristics			
Age (years)	50 [38–53]	48 [38–53]	0.9
Smoker (*n*, %)	9 (41%)	7 (44%)	0.2
Time on mechanical ventilation (days)	3 ± 2	4 ± 2	0.02
P/F (mmHg)	311 ± 157	337 ± 107	0.5
PaO_2_ with FiO_2_ 0,4 (mmHg)	125 ± 52	125 ± 29	0.9
P/F > 350	6 (38%)	11 (50%)	0.5
Cause of death			
Cerebral hemorrhage	6	16	
Anoxic brain injury	3	3	0.7
Trauma	4	1	
Other	3	2	

List of abbreviations: EVLP, *ex vivo* lung perfusion; ECMO, extracorporeal membrane oxygenation; LTx, lung transplantation; CPB, cardiopulmonary bypass; COPD, chronic obstructive pulmonary disease; P/F PaO_2_/FiO_2_ ratio; PF/100.

Among the 38 transplanted patients, there was not enough evidence to show that the patients included in the no-CytoSorb^®^ group had more frequently severe-grade 3 PGD (with the definition and grading by the report of the ISHLT in 2016 [14], retrospectively adopted for all the patients) if compared to the patients in the CytoSorb^®^ group both at arrival in ICU and at 72 h after transplant [11 (50%) vs. 3 (19%) pts, and 6 (28%) vs. 1 (6%), respectively], and needed more frequently post-transplant VV-ECMO [8 (36%) vs. 4 (25%) pts]. The patients included in the no-CytoSorb^®^ group showed a higher in-hospital mortality [5 (23%) vs. 0 pts, *p* = 0.03] and 1-year death [8 (36%) vs. 0 pts, *p* = 0.01] (Supplementary Table S1).

## Discussion

The present study shows our experience on consecutive unselected lung grafts treated with the EVLP technique before their assessment for transplant suitability. The analysis has been focused on the feasibility and safety of the use of an adsorbent device during EVLP.

The mechanism of action of EVLP is still not completely understood: the use of a hyper-oncotic perfusion solution in suboptimal grafts counteracts lung parenchyma fluid overload, thus allowing the recovery of an optimal pulmonary function. However, a more complex mechanism of action involving the fragile balance of inflammatory and anti-inflammatory response can also be supposed. EVLP may act as a “purification” system from potentially toxic molecules such as inflammatory mediators related to the static cold ischemic storage of lung grafts before EVLP [15–19].

During the “cold ischemic period”, potentially harmful events—such as reactive oxygen species formation, sodium pump inactivation, intracellular calcium overload, iron release and cell death—may occur. These phenomena can promote the upregulation of adhesion molecules and the release of pro-inflammatory mediators with recruitment and activation of donor or recipient leukocytes after reperfusion [19]. The inflammatory response associated with the release of pro-inflammatory cytokines may play a pivotal role in the development of PGD [20]. To date, little is known whether the inflammatory response can be contrasted during EVLP.

In our series, a significant increase in cytokines levels has been found during perfusion despite the use of Cytosorb^®^. Same results have been registered by Sadaria et al. [21] who investigated cytokine expression profile and histologic effects in human donor lungs undergoing prolonged normothermic EVLP. Moreover, inflammatory response during EVLP has been associated both to the final result of the EVLP and to the pulmonary function after transplant [22].

The inflammatory cytokine profile expression after lung transplantation has been studied by various groups. De Perrot et al. [9] in Toronto explored cytokine expression in transplanted lungs during cold ischemia and at different timepoints during reperfusion. This study demonstrated that tumor necrosis factor (TNF)-α, interferon (IFN)-γ, IL-10, IL-12, and IL18 were elevated during ischemia, whereas IL-8 was predominantly elevated after reperfusion. In another study, given the anti-inflammatory effects of IL-10, Cypel et al. [23] tried to apply this effect during EVLP. After the delivery of an adenoviral vector encoding human IL-10 during EVLP in the airways, the authors showed significant improvement in pulmonary function in comparison with those lungs undergoing EVLP alone.

Unfortunately, the parameters commonly used for the physiological assessment during EVLP do not allow to precisely predict pulmonary function after transplantation. Inflammation burden during EVLP has been proposed to predict donor related lung injury after transplant [24] and persistence of severe-grade 3 PGD at 72 h seems to be associated with higher levels of IL 6, IL1b and MCP1 in the perfusate [25].

However, so far EVLP is more commonly used to preserve and to evaluate grafts than as an effective strategy to obtain a real reconditioning. On the other hand, EVLP represents a reliable platform to be used in order to repair injured dysfunctional grafts. Among other solid organs, the lungs are unique because of its dual access system and both bronchial or vascular route can be used for direct intervention. The EVLP phase may potentially allow the administration of the most effective therapies based on the specific causes of lung injuries avoiding systemic toxicity. In particular, the reduction of the inflammatory storm can be theoretically addressed during EVLP.

In overall perfusions, despite a significant lower concentration of cytokines at the end of EVLP in the grafts perfused with Cytosorb^®^, deltas from T0 and Tf between Cytosorb^®^ and no-Cytosorb^®^ group are decreased only for IL10 and not for IL6 and MCP1. However, it must be taken into account that indication to EVLP is a poor graft gas exchange due to several reasons in which inflammation can play a major role and this process can be reversible or not. In case of not reversible cause of graft dysfunction and/or inflammatory state (i.e., misdiagnosed pneumonia or irreversible ventilatory lung injury or primary pulmonary disease) the EVLP remains useless to rehabilitate marginal or initially rejected grafts regardless the use of Cytosorb^®^. In these situations, the inflammatory cascade is maintained during perfusion and the cytokines removal may not be effective due to the continue production and release of inflammatory products. Moreover, the duration of EVLP is clinically driven and it is based on clinical parameters (always unrelated with inflammation) collected during the perfusion suggesting the utility or futility of the EVLP. This creates a different length of duration of the treatment. As a matter of fact, median duration of EVLP was 4 (IQR 3–4) and 5 (IQR 4–6) hours in the rejected and transplanted groups, respectively (*p* < 0.01) and IL6 concentrations higher in the rejected grafts in comparison with transplanted grafts (IL6 @*T0* 4.2 IQR 3.3–4.5 vs. 3.2 IQR 2.6–3.6, *p* = 0.01, respectively). This means that, based on clinical evaluation, the rejected grafts are more “inflamed” and less responsive to EVLP with or without Cytosorb^®^ and the reconditioning therapy is shorter because the futility of EVLP becomes evident earlier. Conversely, the transplanted group is more homogenous for the duration of EVLP and cytokines concentrations at the beginning of perfusion. In this cohort the effect of cytokines’ absorption can be more visible: same T0 concentration or even worse level in the Cytosorb^®^ group, same duration of treatment, less cytokines concentration at the end of the perfusion and lower deltas.

The main finding of our initial clinical series is that the reduction of the level of inflammatory mediators can be effectively achieved using a porous polymer beads adsorption during EVLP with the use of CytoSorb^®^ in the clinical setting of lung transplantation and this represents the first experience reported in man so far.

As a matter of fact, the impact of cytokines removal during EVLP has been investigated in the animal model only, both on normal or injured grafts and never in man. In 2010 the Japanese group of Kakishita and coworkers [26] tested for the first time an adsorbent membrane (Lixelle S-35) during EVLP on normal swine lungs. The EVLP was run for 12-h. The filter was laterally attached to the circuit in order to remove pro-inflammatory cytokines from perfusate. The authors showed a significant reduction of TNF-α and IL-8 levels without any impact on pulmonary function suggesting that cytokines removal is effective and safe. Iskender et al. [27] in 2017 hypothesized that cytokine filtration would improve lung function through the clearance of inflammatory mediators during prolonged EVLP. Ten pig lungs were stored at 4°C for 24 h and randomly divided into two groups according to the use or not of the filter added to the EVLP circuit. From their analysis, continuous filtration through beads has been shown to decrease cytokines concentration with a better pulmonary function during EVLP. Moreover, the post-transplant beneficial effects [28] of perfusate adsorption during EVLP have been studied in an animal model of injured grafts showing a more preserved post-transplant graft function in those grafts treated with EVLP plus CytoSorb^®^.

Our study suffers from both conceptual and methodological limitations. CytoSorb^®^ acts as a not selective filtering membrane according to the dimensions of molecules and porous beads. Mechanical removal depends on concentration and molecular weight (up to 50 KDa) of the mediators, therefore both inflammatory and anti-inflammatory cytokines are removed. It can be speculated that however, the removal of pro-inflammatory mediators overcomes the potential negative impact of anti-inflammatory cytokines removal. Moreover, the role of the ratio between pro and anti-inflammatory mediators could be investigated. From a methodological perspective, our results come from a no-randomized retrospective series and potentially confounding factors may jeopardize our clinical findings. Many factors (both related to donor and recipient) during all the phases of transplant (from organ retrieval, *ex-vivo* perfusion and implantation) may interact each other in the definitive decision-making process. Moreover, the two groups refer to a different “historical” period: the no-CytoSorb^®^ cohort refers also to the very beginning of EVLP program with an intrinsic learning curve phase in terms of indication, management and assessment of grafts treated with EVLP. It should be noticed that the two cohorts of transplanted patients are similar but with some statistically significant differences. For example, the need of CPB was higher in the no-CytoSorb^®^ group even if the no-CytoSorb^®^ recipients were younger and receiving more frequently a bilateral transplant (the latter are well-known positive prognostic factors). Regarding recipients’ characteristics no-CytoSorb^®^ group received a graft from donors with a longer mechanical ventilation time although this statistical difference seems insignificant from a clinical point of view (3 vs. 4 days). Finally, the relatively small sample size does not allow a deeper statistical analysis reducing the possibility to draw robust conclusions. However, our first aim was only to evaluate the safety and efficacy of cytokines reduction in the clinical setting. Considering the mean values of cytokines levels (IL-6 log10, IL-10 log10, MCP1 log10, GCSF log10) at *Tf* between the Cytosorb^®^ and no-Cytosorb^®^ groups, their standard deviation and the total sample size, with an alpha error of 0.05, our study power is ≥0.95. The power of the mean difference between base 10 logarithm of IL-6, IL-10 and GCSF levels at *Tf* and *T0* in the transplanted population between the Cytosorb^®^ and no Cytosorb^®^ groups, considering their standard deviation and the total sample size, with an alpha error of 0.05 is ≥0.93 and 0.45 for MCP1.

The clinical impact of cytokines adsorption must be further validated in more rigorous, prospective, randomized clinical trials. However, our analysis refers to a consecutive lung transplant series in a medium-volume center and it can be considered a representative picture of daily clinical practice given the limited number of lung transplants and even fewer procedures of EVLP run worldwide. Clinical scenario of lung transplantation is changing and graft perfusion techniques play an important role.

EVLP not only represents a reliable platform to evaluate and preserve graft before transplant but it can be potentially used to manipulate organs and to achieve a proper reconditioning process. Inflammatory response has been shown to have a central role on graft function after transplant and an active treatment using removal strategies of cytokines during perfusion is very attractive.

## Data Availability

The raw data supporting the conclusion of this article will be made available by the authors, without undue reservation.
