# Supplementary material for: Cytokines Removal During Ex-Vivo Lung Perfusion: Initial Clinical Experience
Source: Transpl Int. 2023 Aug 14;36:10777. doi: 10.3389/ti.2023.10777 (PMC10460908; doi:10.3389/ti.2023.10777)
Supplement: Supplementary file 1 [file Table1.docx]

**Table 1S. Comparison of post-transplant outcomes using grafts after Ex-Vivo Lung Perfusion with and without CytoSorb®**

| ***Post-operative outcomes*** | | | | |
| --- | --- | --- | --- | --- |
|  | ***Cytosorb***  ***(n=16)*** | ***No Cytosorb (n=22)*** | ***P value*** |  |
| Severe-grade 3 PGD at ICU arrival (n, %) | 3 (19%) | 11 (50%) | 0.08 |  |
| Severe-grade 3 PGD after 72 hours (n, %) | 1 (6%) | 6 (28%) | 0.20 |  |
| Need of ECMO after LTx | 4 (25%) | 8 (36%) | 0.50 |  |
| In-hospital death (n, %) | 0 | 5 (23%) | 0.03 |  |
| Death after 1 year (n, %) | 0 | 8 (36%) | 0.01 |  |
| ICU length of stay (days) | 7 [4-12] | 11 [6-22] | 0.20 |  |
| Hours on mechanical ventilation | 65 [24-120] | 88 [31-216] | 0.37 |  |

**List of abbreviations:** EVLP ex vivo lung perfusion; PGD, primary graft dysfunction; ICU, intensive care unit; LTx, lung transplantation
